# The “treatment gap” in global mental health reconsidered: sociotherapy for collective trauma in Rwanda

**DOI:** 10.3402/ejpt.v6.28706

**Published:** 2015-11-19

**Authors:** Stefan Jansen, Ross White, Jemma Hogwood, Angela Jansen, Darius Gishoma, Donatilla Mukamana, Annemiek Richters

**Affiliations:** 1 Center for Mental Health, College of Medicine and Health Sciences, University of Rwanda, Kigali, Rwanda; 2 Mental Health and Well-being, The University of Glasgow, Glasgow, UK; 3 No affiliation; 4 Community Based Sociotherapy Program, Kigali, Rwanda; 5 Department of Public Health and Primary Care, Leiden University Medical Center, Leiden, The Netherlands; 6 Amsterdam School for Social Science Research, University of Amsterdam, Amsterdam, The Netherlands

**Keywords:** Collective violence, community, global mental health, trauma, treatment gap, Rwanda, sociotherapy

## Abstract

**Background:**

The “treatment gap” (TG) for mental disorders refers to the difference that exists between the number of people who need care and those who receive care. The concept is strongly promoted by the World Health Organization and widely used in the context of low- and middle-income countries. Although accepting the many demonstrable benefits that flow from this approach, it is important to critically reflect on the limitations of the concept of the TG and its implications for building capacity for mental health services in Rwanda.

**Objective:**

The article highlights concerns that the evidence base for mental health interventions is not globally valid, and problematizes the preponderance of psychiatric approaches in international guidelines for mental health. Specifically, the risk of medicalization of social problems and the limited way in which “community” has been conceptualized in global mental health discourses are addressed. Rather than being used as a method for increasing economic efficiency (i.e., reducing healthcare costs), “community” should be promoted as a means of harnessing collective strengths and resources to help promote mental well-being. This may be particularly beneficial for contexts, like Rwanda, where community life has been disrupted by collective violence, and the resulting social isolation constitutes an important determinant of mental distress.

**Conclusions:**

Moving forward there is a need to consider alternative paradigms where individual distress is understood as a symptom of social distress, which extends beyond the more individually oriented TG paradigm. Sociotherapy, an intervention used in Rwanda over the past 10 years, is presented as an example of how communities of support can be built to promote mental health and psychosocial well-being.

According to data from the Global Burden of Disease Study 2010, 7.4% of the global burden of disease is attributed to mental health, and neurological and substance use disorders (Whiteford et al., 2013). Although 80% of the world's population live in low- and middle-income countries (LMIC; Saxena, Thornicroft, Knapp, & Whiteford, 2007), >90% of mental health resources are located in high-income countries (WHO, 2005). It is estimated that in LMIC between 76 and 85% of people with severe mental disorders receive no treatment for their mental health conditions (Demyttenaere et al., 2004). This has been referred to as the “treatment gap” (TG), that is, the difference between the number of people with mental health disorders and the number of those people who are able to access appropriate services (Kohn, Saxena, Levav, & Saraceno, 2004). The concept of the TG has been used to highlight the ethical and moral imperative to take action for mental health by LMIC governments, non-governmental organizations, and funding agencies.

In recent years, the Movement for Global Mental Health has emerged to highlight mental health as an under-recognized issue in LMIC, whilst also working to generate and evaluate ideas to address inequalities and inequities in mental health provision across the globe (Lancet Global Mental Health Group, 2007; Patel et al., 2011). In particular, the concept of the TG has also created impetus and momentum for the publication of the *Mental Health Atlas* (WHO, 2005, 2011), which maps available health resources for mental health. Importantly, the concept of the TG has been central to the publication of consensus statements, such as the *Grand Challenges in Global Mental Health* (Collins et al., 2011), which were produced following consultation with over 400 experts from across the globe.

In an attempt to reduce the TG, the World Health Organization (WHO) has made concerted efforts to build capacity for mental health services in LMIC. This includes the *Mental Health Gap—Action Programme* (mhGAP-AP; WHO, 2010) and the *Mental Health Gap—Intervention Guide* (mhGAP-IG; WHO, 2010). The mhGAP-AP outlines key steps for scaling up mental health services in LMIC, whereas the mhGAP-IG presents integrated management plans and evidence-based guidelines for priority neuropsychiatric conditions including depression, psychosis, bipolar disorder, and epilepsy (Dua et al., 2011). With regard to trauma specifically, the WHO (2013) published the Guidelines for the Management of Conditions Specifically Related to Stress, which serves as an adjunct to the existing mhGAP documents and recommendations for assessment and management of acute stress, posttraumatic stress disorder, and grief and prolonged grief disorder (Tol, Barbui, & Van Ommeren, 2013). More recently, the mhGAP *Humanitarian Intervention Guide* (WHO, 2015) has been published which focuses on the implementation of interventions delivered by non-specialist workers in humanitarian settings.

## Limitations of the “TG” paradigm

Concerns have been raised about the narrow definition that the concept of the TG assigns to what actually constitutes treatment (Bartlett, Garriott, & Raikhel, 2014). It has been suggested that the TG has biomedical connotations and does not take into account the variety of treatment choices that may be widely available to someone suffering from distress or a mental health disorder (Fernando, 2014). Indeed, Ventevogel (2014) highlighted that the mhGAP program (WHO, 2008, 2010) has been criticized for potentially contributing to a narrow medical approach to the alleviation of what could be perceived as psychological and social suffering. There is a growing awareness that social determinants of mental health play a significant role in LMIC where a lot of individual suffering can be attributed to social adversity such as poverty, war, and violence (Tol et al., 2014; Whitley, 2015). As such, the mhGAP approach to addressing the TG stands accused of being symptomatic of an ongoing process of medicalization (Clark, 2014; Ingleby, 2014). Others have highlighted that the mhGAP initiatives are narrowly focused on scaling up services based on those designed and implemented in high-income countries and that this has a risk of causing more harm than good in LMIC (Tol et al., 2014; White and Sashidharan, 2014). Jain and Jadhav (2009) raise the possibility that the scaling up of biomedical interventions in parts of India may serve to stifle help-seeking behavior in people who are averse to these treatments—thereby increasing TG. In addition, practical limitations like the lack of human resources, the reluctance of some practitioners to adhere to recommendations, and the lack of systematic reviews for interventions in LMIC pose important obstacles to realistically implementing the mhGAP agenda (Tol et al., 2014).

Paraphrasing Derek Summerfield, Miller (2014) reflects on the possibility that “scaling up psychiatric services to close the presumed gap in mental health provision extinguishes local ways of expressing and dealing with distress, replacing them with particularly Western ways” (p. 131). The possibility remains that the other forms of support can have a positive impact on mental well-being but are often overlooked because these forms of support are not regarded as legitimate ways of addressing the TG. For example, many people turn to religion and spirituality in times of suffering which can give meaning and purpose to one's experiences (Sax, 2014). Indeed, religious beliefs and religious support can help people cope with a mental illness, support recovery, and reduce mental stress (Heim & Schaal, 2014; Webb, Charbonneau, McCann, & Gayle, 2011). In response to this, Abbo (2011) and Patel (2011) have highlighted how important it is to involve traditional healers in efforts to promote mental health in LMIC.

In an attempt to promote improved mental health and well-being across the globe, the WHO launched the *Comprehensive Mental Health Action Plan 2013–2020* (WHO, 2013). This document is intended to complement rather than replace the mhGAP initiatives and has a global focus rather than being focused exclusively on LMIC (as in the case of mhGAP initiatives). One of the four strategic aims of the plan is to emphasize the importance of “providing comprehensive, integrated and responsive mental health and social care services in community-based settings” (p. 10). It could be argued that the concept of the TG has advocated a particularly individualistic approach to mental health difficulties in LMIC. As a consequence of this, the services that may be available in community settings have tended to be focused on the individual rather than addressing suffering that is experienced collectively by communities. Fernando (2012) proposes that the burden of mental health problems on collectivist societies may be greater than the sum of the burden on the individual members of the community. This can be especially so in the context of “collective traumas” (Audergon, 2004; Somasundaram, 2007, 2010), which can be the consequence of events such as armed conflicts or natural disasters. It has also been suggested that, to date, efforts to bridge the TG have used a very limited conceptualization of “community.” Bemme and D'Souza (2014) suggested that global mental health discourses and initiatives have narrowly conceptualized “community” as a method of service delivery, that is, simply an easier way to get to the individual. Rather than a means of investing resources more effectively (Das & Rao, 2012; Saxena et al., 2007) and/or avoiding the iatrogenic impact of prolonged inpatient stays (Wirshing, Smith, Erickson, Mena, & Wirshing, 2006), we propose that “community” should be promoted as a means of harnessing collective strengths and resources to promote mental well-being. This particularly applies in contexts where community life has been disrupted by collective violence and the resulting social isolation proves to be a major determinant of mental distress. In the next section, we will highlight an innovative form of support called community-based sociotherapy, which has been used in Rwanda to utilize “community” as a resource for helping people to cope with daily social stressors and traumatic past experiences related to a history of political violence.

## Sociotherapy in Rwanda: fostering communities as a resource to promote well-being

As the culmination of a long history of violence, in April 1994, the genocide against the Tutsi erupted in Rwanda. Within a population of 7 million, an estimated 800,000 people were killed in a period of 100 days (Des Forges, 1999). The killings were not only a physical act, but also an act of social violation (Fujii, 2009) that served to destroy the social fabric of life in Rwanda. This was exacerbated by the fact that victims and aggressors lived side by side in the same communities. To this day, the impact of the genocide is visible in the day-to-day life. Mass violence has an impact on individuals as well as on the social ties that exist between individuals. Sociotherapy participants talking about these events have emphasized the sense of isolation that developed following the destruction of trust and safety within their communities as being akin to “life without humanity” (Richters & Kagoyire, 2014). This has been described in the literature as “social death” (Card, 2003). To help facilitate a sense of redress for people in Rwanda, an approach was needed that addressed psychological factors operating at both the individual and the community level.

Sociotherapy was developed in Rwanda to alleviate tensions between people and (ethnic) groups at different levels of society. These tensions had the potential to affect people's mental health and prevent a peaceful family and community life (Richters & Sarabwe, 2014). Sociotherapy was adapted from a model used in clinical settings for refugees in the Netherlands (Richters, Dekker, & Scholte, 2008). Its aim is to build safe, trustful, and supportive group environments to facilitate the alleviation of both individual and social distress (Richters et al., 2008). Contrary to centers for individual trauma counseling or mental health care that tend to be situated away from communities, sociotherapy is delivered in the social contexts where people continue to live. It is these social contexts that often serve as ongoing sources of distress in the aftermath of collective traumas (Ngendahayo & Rutayisire, 2011; Panter-Brick, 2010). A typical example in Rwanda is that genocide survivors and perpetrators are brought together in the space of a sociotherapy group. Obstacles to interpersonal reconciliation and ways to overcome them frequently feature in what is said and not said in these groups. As such, sociotherapy makes peace-building part of the recovery process at both the level of the family and the community (Richters, Rutayisire, & Slegh, 2013; Richters & Sarabwe, 2014). Sociotherapy emerges as an interdisciplinary approach; a way to respond to conflict, focusing on both the improvement of mental well-being and the establishment of peaceful communities.

Since its inception in 2005, approximately 20,000 people have participated in sociotherapy groups across the country. Recently, the program has also been introduced in one prison and in various refugee camps in Rwanda. Sociotherapy participants meet in a group of 10–15 people who live in the same neighborhood and select a place to meet where they feel safe such as a church, the house of one of the group members, a classroom, or the grass in the open air. Groups usually meet weekly for 3 h for a total of 15 sessions and slowly work through the phases of safety, trust, care, respect, new life orientations, and memory (as described in detail in Richters, Rutayisire, & Dekker, 2010). The social space of the group is governed by principles such as democracy, equality, and confidentiality, and the aim is for participants to regain their capacity to relate and connect to others so that they can experience again the vitality of humanity and feel mentally healthy (Richters et al., 2010). Through “learning-by-doing,” people discover for themselves what does and does not work for them in terms of new life orientations. This includes establishing positive relationships with others and creating one's own path towards recovery in connection with group members.

The groups are guided by two facilitators who come from the same neighborhood as the group members. Following well-defined criteria (e.g., a minimum standard of education, the motivation to voluntarily assist others, and the availability to do so), the facilitators are selected among community members by program staff in conjunction with local leaders. The facilitators receive short training courses and are monitored in their daily work by sociotherapy field staff. In collaboration with local leaders, the facilitators identify and invite potential participants to the group. Participants are recruited based on problems and tensions observed in the community. They include people who suffer from emotional distress and/or those who are perceived to live in social isolation. The opportunity also exists for people to self-refer into the groups, and experience has shown that this frequently occurs.

No diagnostic criteria are used in the recruitment process. The primary aim of the meetings is not to treat mental health disorders directly, but to work towards social reconnection. It is hypothesized that improved mental health outcomes are a consequence of this process. Although there is no hard evidence to substantiate this claim, a recent pre- and post-intervention study by Verduin et al. (2014) has demonstrated that sociotherapy establishes a significant increase in both civic participation and mental health. Looking at the same data, Scholte et al. (2011) used the Self-Reporting Questionnaire to measure mental health symptoms. They found that after participating in sociotherapy groups, participants reported significantly fewer mental health symptoms than those in a control group, an improvement that persisted at an 8-month follow-up.

Further evidence has been collected about the efficacy of sociotherapy and the pathways towards recovery through (1) regular monitoring of sociotherapy groups, (2) the collection of significant change stories (Jansen & Richters, 2015; Richters & Kagoyire, 2014), and (3) quantitative and qualitative research (Richters et al., 2013; Scholte et al., 2011).

## Building comprehensive paradigms to assist people with mental and psychosocial distress

The example of sociotherapy in Rwanda provides an important illustration of the limitations of the TG paradigm. This paradigm tends to focus on individual psychological distress. The implicit assumption of the TG paradigm is that it is necessary to identify people with mental health disorders and scale up treatments aimed at addressing the symptoms that the individual is experiencing. However, this approach can narrow the scope of our understanding. It can overlook the crucial element of restoring a disrupted social fabric and reshaping communities in a way that previous social divisions that contributed to the past violence and its related suffering are transcended. In contrast, the sociotherapy approach is designed to fit with a paradigm that recognizes individual mental suffering as closely linked to difficulties with social relations, and that people's mental health is strongly linked to healthy family and community ties. In this way, sociotherapy approaches the issue of mental distress from a different perspective. In sociotherapy, people are assisted to reflect in dialog with their peers on their daily life problems and psychological and social suffering. They are encouraged to take the initiative in moving towards more productive coping strategies and increasing their problem-solving capacity. This may include choosing to greet their neighbor instead of avoiding him or her, to stop harassing their children and/or spouse, or taking on civic responsibilities.

In contrast to the tendency to focus on task shifting as a method for building capacity for mental health services in LMIC, the manner in which sociotherapy facilitates the development of “communities of support” is an example of what we term a “task-innovation.” Sociotherapy is innovative in the way it uses community as a resource, rather than simply regarding community as a vehicle for accessing greater numbers of individuals. It does not seek to address restrictive notions of addressing TGs that are focused on individuals; instead it aims to explore new avenues of support that foster communities of support.

## Conclusions

The concept of the TG has been instrumental in guiding efforts to address mental health problems across the globe. It encourages stakeholders to focus on mental disorders experienced by individuals and creates an imperative to scale up particular forms of interventions aimed at delivering circumscribed outcomes. However, difficulties also manifest at the community level and have an impact on the social ties that exist between individuals. As such, broader approaches than the concept of the TG alone are required to guide efforts to build capacity for mental health in LMIC. The application of sociotherapy in Rwanda provides an example of a task-innovation that moves beyond notions of the TG (which manifests at the level of the individual) to include the impact that collective trauma has at a community level.
